# Ethnobotanical study on herbal market at the Dragon Boat Festival of Chuanqing people in China

**DOI:** 10.1186/s13002-021-00447-y

**Published:** 2021-03-23

**Authors:** Qinghe Wang, Ling Zhao, Chi Gao, Jiawen Zhao, Zixuan Ren, Yuxiang Shen, Ruyu Yao, Hongxiang Yin

**Affiliations:** 1 School of Pharmacy, Chengdu University of Traditional Chinese Medicine, Chengdu, 61137 China; 2 School of Ethnic Medicine, Chengdu University of Traditional Chinese Medicine, Chengdu, 61137 China; 3 Anshun College, Anshun, 561000 China; 4 Institute of Medicinal Plant Development, Chinese Academy of Medical Sciences & Peking Union Medical College, Beijing, 10093 China

**Keywords:** Linguistic group, Ethnobotany, Medicinal plant, Nayong County, Traditional knowledge

## Abstract

**Background:**

The Chuanqing people (穿青人) are a linguistic group native to the Guizhou Province of China, with unique culture and rich knowledge of traditional medicinal plants. Herbal market at Dragon Boat Festival (DBF) plays an important role in the inheritance of traditional medicinal knowledge among the Chuanqing people. This study aims to record the profile of medicinal plants of the Chuanqing people, discuss the dilemmas faced by their inheritance, and propose some strategies for passing down information, which is critical for the inheritance and protection of the Chuanqing people’s traditional medical knowledge.

**Methods:**

Data were collected through key informants and semi-structured interviews and free listing. Collected voucher specimens were identified using by botanical taxonomy method and deposited in the herbarium. Data were analyzed through use-value (UV) and cultural importance index (CI) values. Medicinal plants were compared with the Information System of Chinese Rare and Endangered Plants of the Chinese Academy of Sciences. Results were compared with *the Pharmacopoeia of the People’s Republic of China* (ChP), *the Quality Standard of Traditional Chinese Medicine and National Medicine in Guizhou Province* (QSG), and traditional medicines of Southeast Asian countries.

**Results:**

A total of 102 species from 53 families and 92 genera were recorded, with Orchidaceae and Asparagaceae (six species each), and Berberidaceae and Asteraceae (five species each) as the predominant families. The whole plant (36%) was the most common medicinal part. Decoction (44%) was the most common preparation method. Seventy-one investigated human ailments were grouped into 12 categories. Diseases of the musculoskeletal system (34 mentions) were most frequently mentioned in this study. Moreover, the most frequently used taxon was *Hedera sinensis* (Tobler) Hand.-Mazz. (UV and CI = 0.29). The Chuanqing people’s medicine was highly similar to ChP and QSG. In comparison with Southeast Asian countries’ traditional medicines, except for the same preparation methods, the similarities in terms of medicinal ingredients, plants, and disease treatment were very low.

**Conclusions:**

The herbal market at the DBF is an important platform for exchanging knowledge about the Chuanqing people’s traditional medicinal plants. The Chuanqing people’s traditional medicine is facing many challenges to its inheritance and development. To solve these problems, this study highlights the traditional medicinal knowledge of the Chuanqing people, providing basic data for further research and protection of minority medicine.

## Background

In most developing countries, medicinal plants constitute the main materia medica for 70 to 95% of citizens [1–3]. Because of outdated medical facilities and unaffordable medical expenses, traditional medicinal plants have become the first choice or supplement for medical alternatives in most developing countries [4, 5]. In developed countries, increasing numbers of people are also utilizing traditional medicinal plants to treat diseases. With today’s globalized development, traditional medicine has kept pace with the times, which is of great value for the protection of human health. In 2015, for example, Tu Youyou won the Nobel Prize in Physiology or Medicine for the discovery of artemisinin, an extract from traditional Chinese medicine (TCM) *Artemisia annua* L. In 2018, China’s Tibetan medicinal bathing was listed as Intangible Cultural Heritage. To prevent COVID-19, the National Health Protection Commission of China had written the proprietary Chinese medicine Lianhua Qingwen Capsule and Qingfei Paidu Decoction into the guidelines for clinical diagnosis and treatment. All these cases have shown the important role played by traditional medicine in modern society. At the same time, Chinese ethnic medicines have also attracted the attention of researchers [6–11].

Furthermore, as the value of medicinal plants has risen, the traditional market has become an important source of income for citizens. Many studies of traditional herbal markets have been made conducted, such as the herbal markets in Africa and Europe [12, 13], and Hunan [14, 15], and Yunnan Provinces in China [16, 17]. Herbs collected at the Dragon Boat Festivals (DBFs) in China are considered to be of higher quality than those collected at other times [18]. Consequently, people will take advantage of DBFs to collect herbs for use and sale. In Southwest China, the DBFs have become a unique opportunity for local farmers to exhibit and sell local medical resources. Gatherings at DBFs also represent communication platforms for local medical knowledge and experience. The herbal market at the Chuanqing people’s DBF in Guizhou is just such a typical case.

The Chuanqing people are a native linguistic group with a large population in China. They live mainly in Nayong County and Zhijin County of Guizhou Province [19]. According to the genetic relationship, the Chuanqing people are similar to the south Han, Miao, She, and Tujia ethnicities [20, 21]; it is a community with multiple ethnic groups. Every year, on the fifth day of the fifth month of the Chinese lunar calendar, the Chuanqing people in Nayong County prepare a grand herbal market at the DBF. People trade herbs and share their experiences with using herbs and treating diseases at the DBF herbal market. The herbal market has thus become an integral part of the medical culture of the Chuanqing people. This spontaneous traditional activity plays an important role in the inheritance and protection of local traditional medicinal knowledge and sustainable development.

Thus far, there has been a lack of ethnobotanical research on the traditional medicinal plant knowledge of the Chuanqing people in China. Therefore, based on the theories and methods of ethnobotany, this study investigated the medicinal plants of the Chuanqing people in Guizhou to answer three questions: (i) what is the profile of the Chuanqing people’s traditional medicinal knowledge? (ii) what are the differences and similarities between their traditional medicinal plants and *the Pharmacopoeia of the People’s Republic of China* (ChP) [22], *the Quality Standard of TCM and National Medicine in Guizhou Province* (QSG) [23], and traditional medicines of Southeast Asian countries? and (iii) what are the dilemmas and problems faced by the Chuanqing people related to the inheritance and development of traditional medicine knowledge and practices?

## Methods

### Location of the study site

**Fig. 1:**
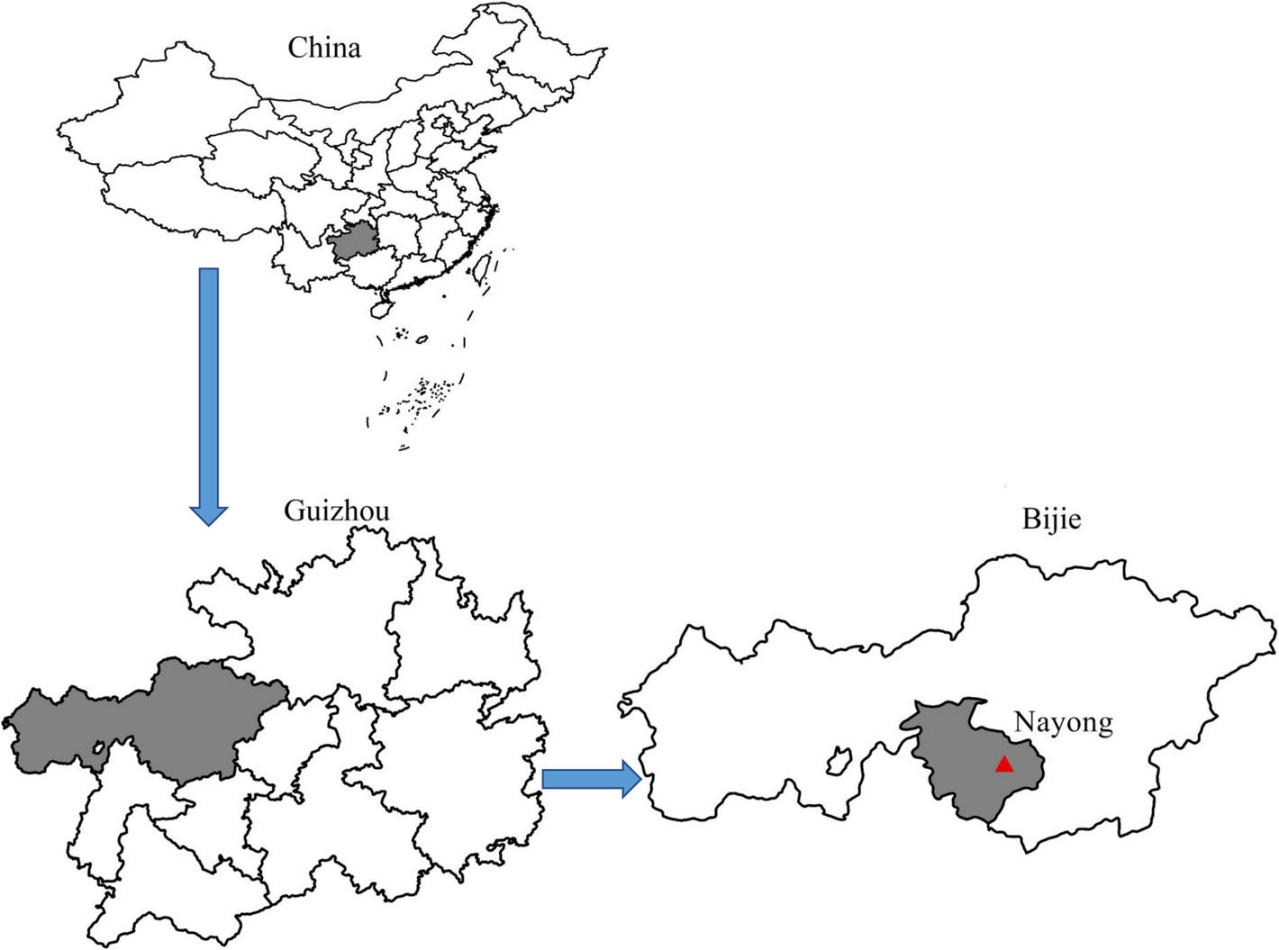
The location of Nayong County, Guizhou Province, China

The study was conducted in Nayong County of Guizhou Province, China (105° 38′ 04″ E and 27° 05′ 54″ N) (Fig. 1). The area has a wide karst landform, which is the transition zone from the Yunnan-Guizhou Plateau to the Wumeng Mountain area. The elevation ranges from 1050 to 2476 m, with an average elevation of 1685 m. The mean annual temperature is 13.7 °C, the mean sunshine duration is 1346.3 h, and the mean annual precipitation is 1203.0 mm. According to official data, Nayong County is a vegetation transition zone and has a northern subtropical humid monsoon climate. The vegetation of the karst area is composed of evergreen broad-leaved forest, evergreen deciduous mixed forest, and deciduous broad-leaved forest [24]. The vegetation is luxuriant, and the forest coverage rate is 47.05%. A total of 1857 plant species from 277 families and 772 genera, in addition to 174 species of wild vertebrates from 56 families and 26 orders, have been recorded in Nayong County. Nayong County is rich in biodiversity; it has a provincial dove tree nature reserve, which is home to rare animals and plants, such as *Tetracentron sinense* Oliv., *Prionodon pardicolor* Hodgson, and *Tylototriton kweichowensis* Fang and Chang [25, 26]. This diversity is conducive to the survey of medicinal ethnobotany and specimen collection. The county is located in the core distribution area of the Chuanqing people. The traditional customs and habits of the Chuanqing people are well preserved. The ethnic characteristics of traditional culture and medical knowledge are distinct and representative. The location of the herbal market at the DBF is centered on the Qianwanjia Agriculture Trade Fairs and extends to two streets, Xinjie Road and Gongmao Road in Nayong County.

### Ethnobotanical data collection

This survey was conducted from June 2018 to June 2019 at the DBF. In this survey, 52 informants were investigated, of whom 33 were males and 19 were females, aged 25 to 80 years, with an average age of 56 years. The medicinal materials sold included one to 25 kinds per informant, 90% of which were wild medicinal materials, obtained mainly through self-collection. Key informant interviews, semi-structured interviews, and free listing were used to obtain information about the ages of the vendors, the names of the medicinal materials, the medicinal parts, the preparation methods, and the functions, indications, sources and collection methods of the medicinal materials (Appendix). After collecting the basic information, the medicinal materials were bought from the vendors and used as specimens. Each exsiccata was identified according to the *Flora of China* [27] and *Flora of Guizhou* [28]. The plant families and species followed the *World Flora Online* (http://www.worldfloraonline.org.), which was used to provide a uniform nomenclature after identification. All the voucher specimens were identified by Hongxiang Yin, an Associate Professor of the Chengdu University of TCM and Yuxiang Shen, an Associate Professor of the Anshun College. The voucher specimens were preserved at the Specimen Center of Chengdu University of TCM (CDCM). At the same time, the collected plant information was compared with the ChP [22], QSG [23], and the traditional medicines in the countries of Southeast Asia. The protected status of the collected medicinal plants was identified by the Information System of Chinese Rare and Endangered Plants [29] of the Chinese Academy of Sciences.

### Data analysis

The quantitative statistical indexes of ethnobotany were calculated by Microsoft Excel 2010, including the use-value (UV) and cultural importance index (CI). According to the International Classification of Primary Care (ICPC-2, http://www.who.int/classification/icd/adaptations/icpc2/en/), 71 diseases of the Chuanqing people in Nayong County were classified into 12 categories. The UV of a medical plant species, a quantitative parameter that demonstrates the relative importance of species known by local people, was also calculated as follows:

$$ \mathrm{UV}=\sum \frac{\mathrm{UP}}{n} $$

where UP refers to the number of mentions per species by each informant and *n* is the total number of informants [30].

The CI was used to indicate the spread of the use (number of informants) of each species as well as to determine the diversity of uses.

$$ {\mathrm{CI}}_{\mathrm{S}}=\sum \limits_{u={u}_1}^{u_{\mathrm{NC}}}\sum \limits_{i={i}_1}^{{\mathrm{i}}_N}\frac{{\mathrm{UR}}_{ui}}{N} $$

where *N* is the total number of informants and NC is the total number of use categories. CI is the sum of the proportion of informants who mentioned each of the use categories for a given species. A higher CI value indicates more uses of a species [31].

## Results

### Age and gender structure of the mastery of medical knowledge

**Fig. 2:**
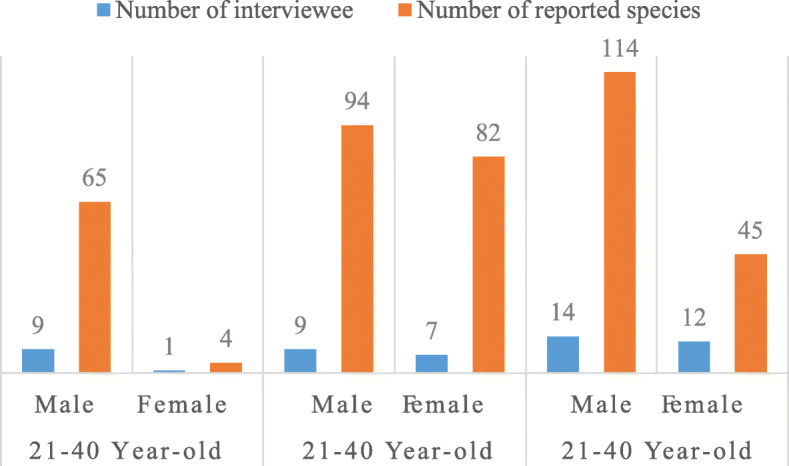
The demographics of interviewees grouped by gender and age, with number of reported species

According to the survey, the number of medicinal materials that were mastered by men was much higher than that mastered by women (Fig. 2). The data showed that 299 herbs were provided by men, whereas only 128 herbs were provided by women, less than half of that provided by men. Additionally, men aged 61–80 years provided the most medicinal materials, whereas men aged 21–40 years provided the least (Fig. 2). Women aged 41–60 years provided the greatest amount of medicinal materials, whereas women aged 21–40 years provided the least (Fig. 2). The knowledge of medicinal plants of the Chuanqing people was mainly mastered by middle-aged and older males (aged 41–80 years).

### Taxonomic characteristics of the medicinal plants

**Fig. 3:**
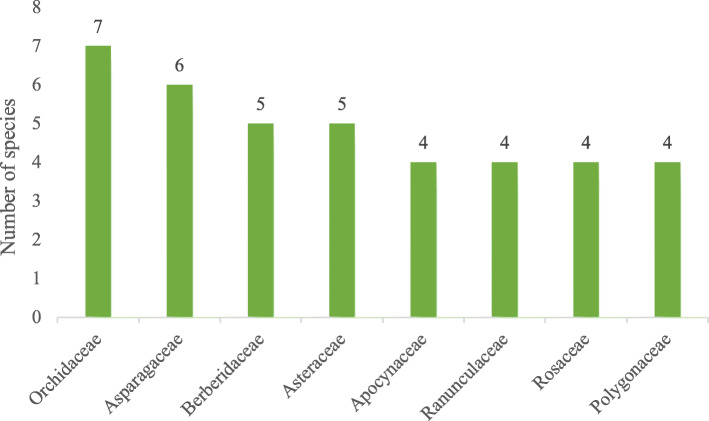
The dominant medicinal plant families and number of species at the herbal market of the DBF

A total of 102 medicinal plant species belonging to 92 genera and 53 families were provided by the Chuanqing people (Fig. 3). The dominant families of the Chuanqing people’s medicinal plants were Orchidaceae and Asparagaceae (six species each), including species such as *Bletilla striata* (Thunb.) Rchb.f., *Reineckea carnea* (Andrews) Kunth, and *Asparagus filicinus* Buch.-Ham. ex D.Don. Following by Berberidaceae and Asteraceae (five species each), including species such as *Senecio analogus* DC. and *Dysosma delavayi* (Franch.) Hu., and Apocynaceae, Ranunculaceae, Rosaceae, and Polygonaceae (four species each). The remaining families were represented by three or fewer entities.

### Analysis of medicinal parts

**Fig. 4:**
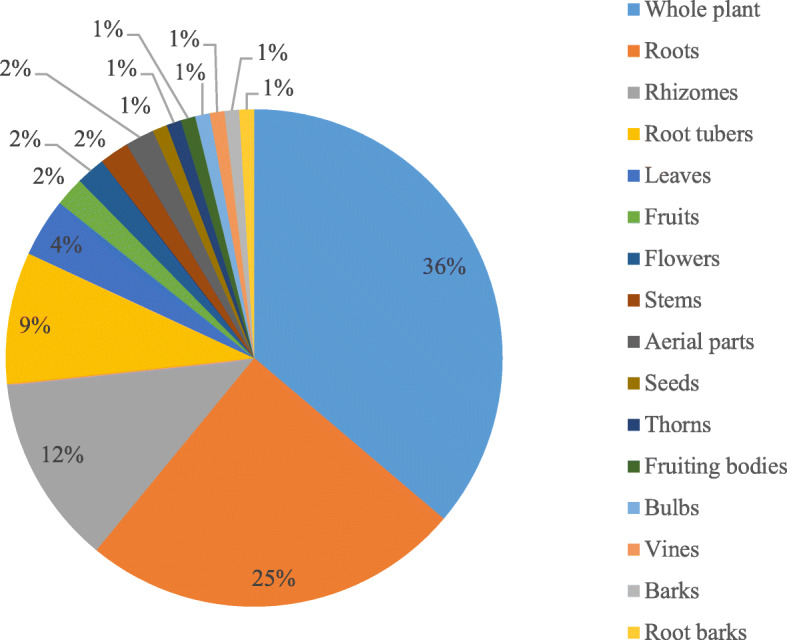
The proportion of medicinal parts at the herbal market of the DBF

Sixteen parts of medicinal plants were used by the Chuanqing people, of which the whole plant was the most common (36%), such as *Dendrobium catenatum* Lindl. and *Taraxacum mongolicum* Hand. - Mazz., etc. This was followed by roots (25%), rhizomes (12%), root tubers (9%), leaves (4%), fruits (2%), flowers (2%), stems (2%), aerial parts (2%) and others (7%) (Fig. 4). The proportion of underground parts that were used as medicinal parts reached 46%.

### Preparation methods

**Fig. 5:**
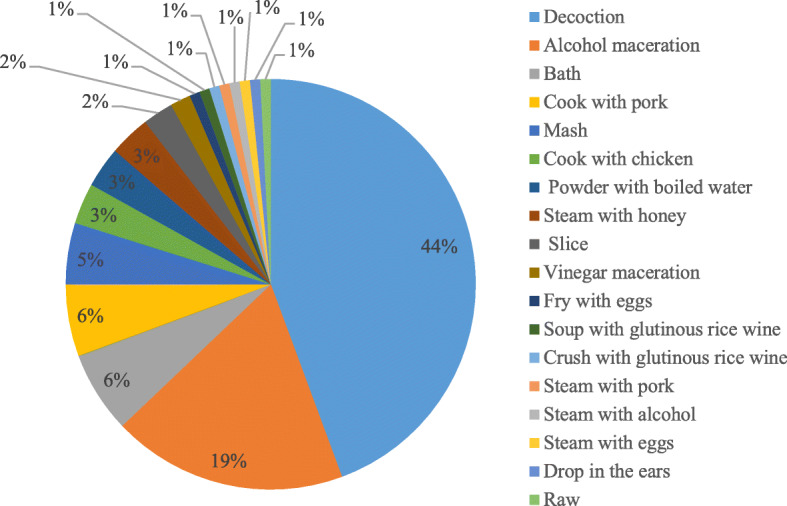
The proportion of medicinal uses at the herbal market of the DBF

Eighteen preparation methods of the Chuanqing people were recorded. Decoction (44%) was the most commonly used preparation method, as observed for *Disporopsis fuscopicta* Hance, *Verbena officinalis* L., and *Polygonum aviculare* L. This was followed by alcohol maceration (19%), bath (6%), cooked with pork (6%), mashed (5%), cooked with chicken (3%), powdered with boiled water (3%), steamed with honey (3%), sliced (2%), vinegar maceration (2%), and others (6%) (Fig. 5). Additionally, there were some special medical methods used by the Chuanqing people, such as souping with glutinous rice and firing with eggs.

### Functions and indications

According to the International Classification of Primary Care (ICPC-2, https://www.who.int/classifications/icd/adaptations/icpc2/en/), the herbs sold at the herbal market were used to treat 71 human ailments, which were divided into 12 categories (Table 1). Most medicinal materials were used to treat diseases of the musculoskeletal system (34 mentions), followed by diseases of the digestive system (18 mentions), certain infectious and parasitic diseases (17 mentions), and diseases of the urinary and genital system (16 mentions), diseases of the respiratory system (15 mentions). The remaining categories of aliments were represented by one to five mentions.

**Table 1:** The number of categories of ailments

Category	Number
Musculoskeletal system	34
Digestive system	18
Certain infectious and parasitic diseases	17
Urinary and genital system	16
Respiratory system	15
General	5
Circulatory system	5
Neurological system	3
Blood and blood-forming organs immune system	2
Ear	2
Pregnancy and childbearing	1
Eye	1

### Analysis of the UV and CI values of medicinal plants of the Chuanqing people

The UV of medicinal plants used by the Chuanqing people ranged from 0.02 to 0.29, whereas many species had low UV and CI values. The UV values of 10 medicinal plants (UV = CI > 0.10) were high (Appendix); the highest UV and CI values were calculated for *Hedera sinensis* (Tobler) Hand.-Mass. (UV and CI = 0.29), *Aconitum carmichaelii* Debeaux, *Plantago major* L., *Persicaria capitata* (Buch.-Ham. ex D.Don) H., *Paris polyphylla* Sm, and *Potentilla discolor* Bunge (UV and CI = 0.13) and *Geum aleppicum* Jacq. (UV and CI = 0.12), and *Artemisia argyi* H.Lév. & Vaniot, *Epimedium acuminatum* Franch., and *Lysimachia paridiformis* var. *stenophylla* Franch. (UV and CI = 0.10).

### Analysis of the species with rare and endangered status

A total of 9 medicinal plants were recorded in the Information System of Chinese Rare and Endangered Plants (Table 2), and nine species were recorded as nationally protected plants. Among them, four species were protected by the Convention on International Trade of Endangered Species of Wild Fauna and Flora (CITES), and five species were recorded in the International Union for Conservation of Nature (IUCN), including least concern (one mention), near threatened (one mention), and vulnerable (three mentions) species. Five medicinal species were endemic to China.

**Table 2:** Records of the information system of Chinese rare and endangered plants

Scientific name	National protection	CITES	IUCN	Distribution area
*Magnolia officinalis* Rehder & E.H.Wilson	(II)		NT	Only in China
*Rhodiolayun nanensis* (Franch.) S. H. Fu	(II)		LC	Only in China
*Pleione yunnanensis* (Rolfe) Rolfe	(II)	II	VU	
*Cibotium barometz* (L.) J. Sm.	(II)	II		Only in China
*Aristolochia tuberosa* C. F. Liang et S. M. Hwang	(II)		VU	Only in China
*Paris polyphylla* Sm	(II)			
*Citrus cavaleriei* H. Lév. ex Cavalier	(II)			Only in China
*Taxus wallichiana* var. *chinensis* (Pilg.) Florin	(I)	II	VU	
*Gastrodia elata* Blume.	(II)	II		

*NT* near threatened, *LC* least concern, *VU* vulnerable

### Comparison of the Chuanqing people’s medicine with the ChP [22], QSG [23], and traditional medicines in Southeast Asian countries

Compared with the ChP and QSG, 39 medicinal plants (38%) were documented by the ChP, 31 (30%) were documented by the QSG, and 11 species were recorded in the ChP, the QSG, and the Chuanqing people’s medicine simultaneously (Appendix). These plants were *Aconitum carmichaelii* Debeaux, *Cynanchum paniculatum* (Bunge) Kitag. ex H.Hara, *Geum aleppicum* Jacq., *Gleditsia sinensis* Lam., *Iris tectorum* Maxim, *Ligusticum striatum* DC., *Lysionotus pauciflorus* Maxim., *Paris polyphylla* Sm, *Reynoutria multiflora* (Thunb.) Moldenke, *Sanguisorba officinalis* L., and *Tinospora sagittata* Gagnep.

The traditional medicinal knowledge of the Chuanqing people was compared with findings studies recently conducted in Thailand, Laos, Vietnam, and Myanmar. The differences in the dominant families, medicinal parts, preparation methods, and diseases of traditional medicinal plants were analyzed as follows (Table 3). The results showed that Leguminosae was the dominant family in these four countries, whereas Asparagaceae and Orchidaceae were the most commonly used families by the Chuanqing people. Leaves were the most common medicinal part of the plant used in Thailand, Vietnam, and Myanmar, and roots and rhizomes were the most common medicinal part in Laos, whereas the whole plant was the most common medicinal part used by the Chuanqing people. Decoction was the most common preparation method in these four countries and the Chuanqing people. In terms of disease treatment, digestive system diseases were the most common in Thailand, Laos, and Myanmar, whereas eye diseases were the most common in Laos, and musculoskeletal system diseases were the most common in the Chuanqing people.

**Table 3:** Comparison of traditional medicine between Chuanqing and Southeast Asian countries

Country	Families	Medicinal parts	Preparation method	Diseases	References
Thailand	Leguminosae, Asteraceae	Leaves, stem	Decoction	Digestive system, infections, nutritional disorders	[32–35]
Laos	Leguminosae, Zingiberaceae	Roots and rhizomes, woody part of plants	Decoction	Gastrointestinal conditions, gynecological conditions, and sexually transmitted diseases skin affections	[36–38]
Vietnam	Asteraceae, Leguminosae	Leaves, roots	Decoction	Eye diseases, musculoskeletal disorders, endocrine/metabolic and nutritional disorders	[39, 40]
Myanmar	Leguminosae, Asteraceae	Leaves, roots	Decoction	Digestive system, urological, respiratory	[41–43]

Table 3 lists listed in the table were the two most frequently used families, the two most frequently used medicinal parts, one of the most frequently used preparation methods, and the three most frequently treated diseases in the four countries.

## Discussion

### The knowledge of traditional medicinal plants of the Chuanqing people

Traditional medicinal knowledge of the Chuanqing people was mastered mainly by men aged 61–80 years (Fig. 2). According to the data of the National Bureau of Statistics, in 2010, the illiteracy rate of men in the rural areas was 29%, and that of women was 71%. Men who received more education might develop more knowledge about medicinal plants. Moreover, the family collaboration model in the local Chuanqing people’s areas was “men work outside and women do housework” [44], so men had more opportunities to identify and collect medicinal plants in the field.

The main families of medicinal plants used by the Chuanqing people were the Orchidaceae and Asparagaceae families. First, as one of the most typical karst areas in the world, Guizhou’s unique geographical location and complex natural environment provided suitable conditions for wild orchids [45]. There are 1240 species of orchids in 171 genera in China, of which 343 species of 82 genera were used for medicinal purposes [46, 47]. Additionally, most plants in Asparagaceae, such as *Polygonatum kingianum* Collett & Hemsl. and *Asparagus filicinus* Buch.-Ham. ex D.Don were used frequently because their thick root tubers were attractive to herb collectors. Second, Orchidaceae and Asparagaceae plants such as *Bletilla striata* (Thunb.) Rchb.f., *Gastrodia elata* Blume., and *Polygonatum kingianum* Collett & Hemsl. were widely used in TCM. These plants were traditional Chinese herbal medicines are commonly used by people of Han nationality. Therefore, a large amount of TCM was used by the Chuanqing people as they had long been influenced by the traditional Chinese medical system.

The underground plant parts (the sum of roots, rhizomes, and root tubers) used in the Chuanqing people traditional medicine accounted for 46%. Therefore, why is the proportion of underground parts of medicinal plants used by the Chuanqing people so high? Roots, rhizomes, and tubers were frequently used as medicinal parts in TCM; more than one fourth of the medicinal plants added in over 400 preparations were derived from roots and/or rhizomes [48]. The whole plant, which accounted for 36%, was commonly used for medicinal purposes because the whole plant was easy to obtain and convenient to use by local people.

Decoction (44%) was the most common preparation method used by the Chuanqing people. Decoction was also the most commonly used TCM compound dosage form by traditional Chinese doctors, and it was also the longest and most widely used preparation in the history of China [49]. Since the decoction method was also the most commonly used preparation method for the Chuanqing people, it can be seen again that their medicine has a long history of being influenced by TCM. Alcohol maceration was the second most common preparation method, accounting for 19%. Alcohol maceration was also a traditional Chinese medicinal preparation, with unique curative effects, a convenient preparation method, and wide application [50]; it was thus widely used. The Chuanqing people also had some other special usages, such as making a soup with glutinous rice wine and steaming with eggs.

Most medicinal materials were used to treat diseases of the musculoskeletal system (34 mentions). For instance, *Artemisia argyi* H.Lév. & Vaniot (UV and CI = 0.10), *Hedera sinensis* (Tobler) Hand.-Mazz. (UV and CI = 0.29), and *Lysimachia paridiformis* var. *stenophylla* Franch. (UV and CI = 0.10) were all used to treat rheumatism; *Liparis campylostalix* Rchb.f., *Rhodiola yunnanensis* (Franch.) S. H. Fu, and *Cynanchum inamoenum* (Maxim.) Loes. ex Gilg. & Loes. were used to treat traumatic injuries. One reason might be that, according to statistics, the diseases with the fastest increase in hospitalization and expenses in 2015 in China were musculoskeletal diseases [51]. Moreover, people who lived in humid climates and engaged in agriculture, typically had a variety of musculoskeletal system diseases, such as rheumatism [52], traumatic injuries, and other diseases, as do the Chuanqing people.

### Comparison with ChP, the QSG, and Southeast Asian medicines

The existing studies on the Chuanqing people showed that they were closely related to the Han and local ethnic groups in Guizhou such as the Miao in their social culture [20, 21, 53] or medical research [54]. Eleven medicinal plants were also recorded in the ChP, the QSG, and the Chuanqing people’s medicine simultaneously, suggesting that the diseases treated with medicinal plants by the Chuanqing people were similar to those found among the ChP and the QSG. For example, *Aconitum carmichaelii* Debeaux was used to treat noxious sores and had the function of restoring yang for resuscitation; *Tinospora sagittata* Gagnep. was used to treat neck pain, laryngitis, dysentery, and abdominal pain; and *Ligusticum striatum* DC. was used to relieve pain by people of ChP, QSG, and the Chuanqing people. However, the diseases treated with some medicinal plants of the Chuanqing people were different from those of both ChP and QSG. For example, *Cynanchum paniculatum* (Bunge) Kitag. ex H.Hara was used to relieve pain in ChP and QSG, but it was used to treat gynecopathy in the Chuanqing people’s medicine and some researchers found that it could treat gynecological inflammation disease because of its anti-inflammatory properties [55]. *Gleditsia sinensis* Lam. was used to treat osteodynia, and arthralgia rather than psychiatric disorders in ChP and QSG, and some studies found that it also had analgesic effects [56]. *Lysionotus pauciflorus* Maxim. was first found to treat rheumatism, and *Paris polyphylla* Sm was first found to treat cardiopathy. *Sanguisorba officinalis* L. was first found to treat diarrhea in Chuanqing people; this treatment had been corroborated in the reports about being used to treat diarrhea of humans and livestock [57–59].

Generally, after thousands of years of development, TCM had formed a mature theoretical system, such as “the theory of Four Qi and Five Flavors, the theory of Visceral Manifestation, and the theory of Yin-Yang and Five Elements.” According to our survey, the Chuanqing people’s medicine was still in the stage of summarizing specific knowledge and experience, such as circulated in the form of rhymes.

Southwest China is adjacent to the Southeast Asian countries of Vietnam, Laos, Myanmar, and Thailand [60]. Historically, they had often exchanged cultural practices and medicine. The traditional medicines in Southeast Asian countries were deeply influenced by TCM [61–64]. The Chuanqing people in southwestern China were also influenced by traditional Chinese medicine [65]. Therefore, the Chuanqing people and the people of Southeast Asian countries may have certain similarities in their use of medicinal plants. For instance, decoction was the most common preparation method among them because decocting was the most convenient and simplest method. However, there are also differences in their use of traditional medicinal plants. For example, Leguminosae was widely used in traditional medicines in Southeast Asian countries, whereas Asparagiaceae and Orchidaceae were widely used by the Chuanqing people. The reason for this difference was that Leguminosae was widely distributed in tropical flora and available in these countries [32–41]. The area of southwest China populated by the Chuanqing people is a typical karst landform, with the majority of the plants distributed in Asparagaceae and Orchidaceae [45–47]. Leaves are frequently used in Southeast Asian countries; because this region has a tropical rainforest climate and many evergreen plants grow up there, the leaves are abundant and easy to obtain. However, the areas in Guizhou Province populated by the Chuanqing people have a northern subtropical monsoon climate, with a high altitude (1050 to 2476 m) and four distinct seasons. In autumn and winter, the whole plant and underground parts could be used without leaves. In Southeast Asian countries, the most common diseases treated by traditional plants were diseases of the digestive system. Studies had pointed out that this was mainly related to living conditions, living habits, and sanitation facilities. For example, in the countries of Southeast Asia, people who worked in agriculture and lived in poverty were susceptible to the diseases of the digestive system, such as gastritis and diarrhea [32, 37]. Alcoholism was also a reason for digestive system diseases [41]. The reasons why the Chuanqing people’s medicine mainly treats musculoskeletal diseases were discussed above. The approaches found in Thailand, Laos, Vietnam, and Myanmar regarding the dominant plant families, medicinal parts, and treatment of diseases of traditional medicine were different than those of the Chuanqing people, with few similarities.

### The dilemmas and solution of the Chuanqing people’s traditional medicine culture

The Chuanqing people’s traditional medicinal knowledge was mastered by people aged 61–80 years, leading to the problem of traditional knowledge being concentrated in older members of the community. Additionally, a large number of rural young people have chosen to move to big cities to work and live in recent years, aggravating the problem of the aging population. This demographic development was not conducive to the inheritance and development of the Chuanqing people’s knowledge of traditional medicinal. Furthermore, people’s lifestyles have been changed by the impact of modern industrial civilization; their medical choices have also been altered because of the popularization of modern medicine. These factors have led to the decline of the social recognition of traditional medical knowledge and the decrease of users of traditional medicinal practices, which further endangers the application and protection of this knowledge. It is important to strengthen the collection and protection of local traditional medicinal knowledge, conduct a comprehensive interview with the older generation of ethnic doctors, and collect and document the diagnoses and treatment methods with ethnic characteristics.

In recent years, with the increasing demand for natural medicines, many wild medicinal materials have been plundered without scientific protections or development measures. For example, uprooting the whole plant and underground parts as the most commonly used in traditional medicine was not conducive to the regeneration of wild plant populations. Moreover, we found cases of people selling wild protected animals and plants, such as *Paris polyphylla* Sm, *Taxus wallichiana* var. *chinensis* (Pilg.) Florin, and *Tylototriton kweichowensis* Fang and Chang (listed as vulnerable (IUCN, 2012) and as category II state major protected wildlife in China). This indicated that local, rare, wild plant and animal resources have been destroyed and that legal risks are present in the DBF herbal market. In the face of this situation, the government and non-governmental agencies should strengthen the natural protection of wild species by increasing law enforcement and strengthening the popular science education of local communities. For species with significant economic value, scientific institutions should accelerate scientific research on artificial breeding and cultivation, instead of utilizing wild populations.

Because of the lack of modern scientific and technological means and government guidance, the development of industrialized, modernized planning of the Chuanqing people’s medicine was almost absent. To a certain extent, this situation led to the loss of cultural knowledge of the Chuanqing people’s medicine and the dilemmas of sustainable development. It should be noted that China’s DBF was added to the United Nations Educational, Scientific, and Cultural Organization’s Intangible Cultural Heritage list in 2009. Moreover, many regions have upgraded traditional ethnic medicinal markets at the DBF into well-known cultural tourism products. For example, the DBF medicinal market of the Zhuang in Jinxi County has been selected as the intangible cultural heritage of the Guangxi Zhuang autonomous region [66], and the Pu’er City of Yunnan Province promoted the local DBF herbal market as a “Baicao Gen Food and Cultural Tourism Festival” [16]. These examples provide arguments for passing down and promoting the traditional medical culture of the Chuanqing people.

## Conclusions

This is the first study to document the traditional medicinal knowledge of the Chuanqing people in China. A total of 102 species from 53 families and 92 genera were recorded to treat 71 human aliments, which were divided into 12 categories. Most medicinal materials were used to treat diseases of the musculoskeletal system (34 mentions). A total of nine medicinal plants were recorded in the Information System of Chinese Rare and Endangered Plants. Many plants with high UV and CI values need more attention and further research. There are some differences and connections among the Chuanqing people’s medicine, ChP and QSG. The Chuanqing people and people of the countries of Southeast Asia have many similarities in preparation methods but few low similarities in medicinal families, medicinal parts, and diseases. The DBF herbal market is an important platform for the Chuanqing people to inherit their traditional medicinal knowledge. The Chuanqing people are rich in medicinal plant species, knowledge, and experience, which reflects their own cultural and regional characteristics. The inheritance and development of traditional medicine by the Chuanqing people have faced many difficulties, such as aging, the impact of modern civilization, exhaustion of resources, legal risks, and lack of policy planning. It is, therefore, necessary that more in-depth research on the Chuanqing people’s medicinal plants be conducted; the knowledge of traditional medicinal plants is protected by formulating appropriate policies and practices.

## Data Availability

All data generated or analyzed during this study are included in this published article and its supplementary information files.

## Appendix

*PU* parts used, *MP* method of preparation, *MU* medicinal use, *PR* pharmacopoeia records, when compared with PR, medicinal plants used by *the Pharmacopoeia of the People's Republic of China* (ChP) are marked “A”, medicinal plants used by *the Quality Standard of TCM and National Medicine in Guizhou Province* (QSG) are marked “B”, and similar use is marked “AB”.

**Table 4:** Medicinal plants used by the Chuanqing People

Scientific name	Local name	Family name	PU	MP	MU	Voucher specimen number	PR	UV	CI
*Achyranthes longifolia* (Makino) Makino	Bai Niu Xi	Amaranthaceae	Whole plant	Alcohol maceration	An internal lesion caused by overexertion	NY-045		0.02	0.02
*Aconitum carmichaelii* Debeaux	Hao Zi Tou	Ranunculaceae	Roots	Vinegar maceration	Noxious sore, restoring yang for resuscitation	NY-033	AB	0.13	0.13
*Aconitum sinomontanum* Nakai	Chuan Xin Lian	Ranunculaceae	Rhizomes	Decoction, alcohol maceration	Gastrosia, abdominal pain	NY-001	B	0.04	0.04
*Acorus macrospadiceus* (Yamam.) F.N.Wei & Y.K.Li	Cao Pi Hui Xiang	Acoraceae	Whole plant	Decoction	The pain of cold air in the heart and abdomen, deafness and muteness	NY-047		0.04	0.04
*Akebia trifoliata* (Thunb.) Koidz.	Mu Tong	Lardizabalaceae	Rhizomes	Decoction	An internal lesion caused by overexertion	NY-179	A	0.06	0.06
*Alcea rosea* L.	Hong Qi Hua	Malvaceae	Flowers	Soup with glutinous rice wine	Gynecopathy	NY-041		0.02	0.02
*Amorphophallus konjac* K. Koch	Shan Mo Yu	Araceae	Root tubers	Alcohol maceration, mash	Cervical spondylopathy	NY-103		0.02	0.02
*Anemone rivularis* Buch. -Ham. ex DC.	Ba Ban Hua	Ranunculaceae	Roots	Decoction	Anti-inflammatory	NY-120		0.08	0.08
*Ardisia crispa* (Thunb.) A. DC.	Guo Shan Long	Primulaceae	Roots	Decoction	Tonsillitis	NY-009	B	0.02	0.02
*Arisaema heterophyllum* Blume	Tian Nan Xing	Araceae	Root tubers	Mash	Drying dampness to dominate phlegm	NY-250	A	0.02	0.02
*Aristolochia cucurbitoides* C. F. Liang	Qing Teng Xiang	Aristolochiaceae	Roots	Decoction	Relieve pain	NY-150		0.08	0.08
*Aristolochia tuberosa* C. F. Liang & S. M. Hwang	Zhu Sha Lian	Aristolochiaceae	Roots	Slice	Dysentery, diarrhea, enteritis	NY-101		0.02	0.02
*Artemisia argyi* H.Lév. & Vaniot	Ku Hao	Asteraceae	Rhizomes	Bath	Rheumatism	NY-057	A	0.10	0.10
*Asparagus filicinus* Buch.-Ham. ex D.Don	Tian Men Dong	Asparagaceae	Roots	Decoction	Enrich the blood, suppress cough	NY-258		0.06	0.06
*Aster indicus* L.	Huang Hua Cao	Asteraceae	Whole plant	Alcohol maceration	Abdominal pain, knee pain	NY-028	B	0.04	0.04
*Begonia grandis* subsp. *sinensis* (A. DC.) Irmsch.	Yi Kou Xue	Begoniaceae	Root tubers	Alcohol maceration, powder with boiled water	Wound, hemostasis	NY-017		0.06	0.06
*Begonia palmata* D. Don	Shui Ba Jiao	Begoniaceae	Root tubers	Decoction	Megrim, gastrosia	NY-228	B	0.02	0.02
*Berberis triacanthophora* Fedde	San Ke Ci	Berberidaceae	Rhizomes	Decoction	Abdominal pain	NY-127		0.02	0.02
*Bergenia purpurascens* (Hook.f. & Thomson) Engl.	Ba Da Jin Gang	Saxifragaceae	Roots	Alcohol maceration	Internal lesion caused by overexertion	NY- 006	A	0.04	0.04
*Bidens tripartita* L.	Zhan Dian Zi	Asteraceae	Roots	Mash	Facilitates qi, intestinal leakage	NY-097		0.04	0.04
*Bletilla striata* (Thunb.) Rchb.f.	Bai Ji	Orchidaceae	Roots	Steam with honey	Suppress cough, pulmonary tuberculosis	NY-263	A	0.04	0.04
*Boschniakia himalaica* Hook. f. & Thomson.	Hao Ling	Orobanchaceae	Whole plant	Cook with chicken	Deficiency of the kidney and lumbago	NY-147		0.02	0.02
*Campylandra chinensis* (Baker) M. N. Tamura et al.	Wan Nian Pa	Asparagaceae	Whole plant	Decoction	Injuries from falls, fractures, contusions and strains, internal lesion caused by overexertion	NY-239		0.04	0.04
*Chloranthus henryi* Hemsl.	Si Kuai Wa	Chloranthaceae	Roots	Alcohol maceration	Internal lesion caused by overexertion	NY-075	B	0.04	0.04
*Cibotium barometz* (L.) J. Sm.	Jin Mao Gou Ji	Dicksoniaceae	Rhizomes	Alcohol maceration, cook with pork	Lumbago	NY-025		0.02	0.02
*Cimicifuga japonica* (Thunb.) Spreng.	Mu Qing Gan	Ranunculaceae	Roots	Slice	Abdominal pain	NY-021		0.02	0.02
*Citrus cavaleriei* H.Lév. ex Cavalerie	Ye Gou Gan	Rutaceae	fruits	Decoction	Nourish the lung to arrest cough	NY-148		0.02	0.02
*Codonopsis pilosula* (Franch.) Nannf.	Dang Shen	Campanulaceae	Roots	Cook with chicken	Nourishing	NY-029	A	0.06	0.06
*Corallodiscus lanuginosus* (Wall. ex DC.) B. L. Burtt	Huan Hun Cao	Gesneriaceae	Whole plant	Steam with pork, mash, steam with alcohol	Infantile malnutrition, hemostasis, injuries from falls, fractures, contusions, and strains	NY-090		0.06	0.06
*Curculigo orchioides* Gaertn.	Xian Mao	Hypoxidaceae	Whole plant	Alcohol maceration	Lumbago	NY-013	A	0.02	0.02
*Cynanchum auriculatum* Royle ex Wight	Ge Shan Xiao	Apocynaceae	Roots	Cook with pork, alcohol maceration	Nourishing	NY-106	B	0.08	0.08
*Cynanchum inamoenum* (Maxim.) Loes. ex Gilg. & Loes.	Luan Tou Fa	Apocynaceae	Roots	Decoction	Internal lesion caused by overexertion and cardiopathy	NY-026		0.06	0.06
*Cynanchum paniculatum* (Bunge) Kitag. ex H.Hara	Dui Ye Lian	Apocynaceae	Whole plant	Decoction	Gynecopathy	NY-249	AB	0.06	0.06
*Dendrobium officinale* Kimura & Migo	Shi Hu	Orchidaceae	Whole plant	Cook with pork	Hypertension, suppress cough	NY-180	A	0.02	0.02
*Disporopsis fuscopicta* Hance	Yu Zhu	Asparagaceae	Roots	Decoction	Promote the secretion of saliva or bodily fluid	NY-102	B	0.06	0.06
*Disporum cantoniense* (Lour.) Merr.	Dao Zhu San	Colchicaceae	Roots	Mash	Osteoarthralgia	NY-098	B	0.02	0.02
*Drynaria roosii* Nakaike	Gu Sui Bu	Polypodiaceae	Roots	Decoction, alcohol maceration	Strengthen tendon and bone	NY-121		0.06	0.06
*Dysosma delavayi* (Franch.) Hu	Ba Jiao Lian	Berberidaceae	Whole plant	Vinegar maceration, alcohol maceration	An internal lesion caused by overexertion, rheumatism, carbuncle toxin, gastrosia	NY-205	B	0.06	0.06
*Epimedium acuminatum* Franch.	Tong Si Cao	Berberidaceae	Whole plant	Steam with honey	Tracheitis	NY-072	B	0.10	0.10
*Fagopyrum dibotrys* (D.Don) H.Hara	Ye Qiao Lan	Polygonaceae	Roots	Decoction	Dyspepsia, abdominal pain	NY-176	A	0.02	0.02
*Fallopia multiflora* (Thunb.) Haraldson	He-Shi Wu	Polygonaceae	Root tubers	Decoction	Cavities, strengthen tendons and bone	NY-110	AB	0.08	0.08
*Fallopia denticulata* (C.C.Huang) Holub	Ji Xue Lian	Polygonaceae	Roots	Decoction	Diarrhea	NY-261		0.02	0.02
*Ficus sarmentosa* var. *impressa* (Champ. ex Benth) Corner	Luo Shi Teng	Moraceae	Whole plant	Decoction	Rheumatism	NY-254		0.02	0.02
*Gastrodia elata* Bl.	Tian Ma	Orchidaceae	Roots	Cook with pork	Nourishing	NY-257	A	0.04	0.04
*Gentiana rigescens* Franch. ex Hemsl.	Long Dan Cao	Gentianaceae	Whole plant	Decoction	Abdominal pain	NY-030		0.04	0.04
*Geum aleppicum* Jacq.	Lan Bu Zheng	Rosaceae	Whole plant	Fry with eggs, decoction	Megrim	NY-034	B	0.12	0.12
*Gleditsia sinensis* Lam.	Zao Jiao Ci	Fabaceae	Thorns	Cook with chicken	Osteodynia, arthralgia	NY-109	AB	0.06	0.06
*Habenaria davidii* Franch.	Shuang Shen Cao	Orchidaceae	Roots	Alcohol maceration	Tonify the kidney, strengthen yang	NY- 003		0.06	0.06
*Hedera nepalensis* var. *sinensis* (Tobler) Rehder	San Jiao Feng	Araliaceae	Whole plant	Bath	Rheumatism, postpartum itchy skin	NY-035	B	0.29	0.29
*Hemsleya chinensis* var. *ningnanensis* L.D. Shen & W.J. Chang	Ku Jing Pen	Cucurbitaceae	Root tubers	Powder with boiled water	Relieve pain	NY-166		0.06	0.06
*Hylotelephium erythrostictum* (Miq.) H. Ohba	San Bai Bang	Crassulaceae	Whole plant	Alcohol maceration	Internal lesion caused by overexertion	NY-052		0.04	0.04
*Iris tectorum* Maxim	Sou Shan Hu	Iridaceae	Rhizomes	Slice	Abdominal pain	NY-137	B	0.04	0.04
*Lasiosphaera seu* Calvatia	Ma Pi Bao	Lycoperdaceae	Fruiting bodies	Mash	Pus and sores	NY-157		0.02	0.02
*Ligusticum chuanxiong* S.H.Qiu, Y.Q.Zeng, K.Y.Pan, Y.C.Tang & J.M.Xu	ChuanXiong	Apiaceae	Rhizomes	Steam with eggs, alcohol maceration	Sprain, lumbago	NY-093	AB	0.04	0.04
*Lilium brownii* F.E.Br. ex Miellez	Bai He	Liliaceae	Bulbs	Cook with chicken, steam with honey	Nourish the lung to arrest cough	NY-159		0.02	0.02
*Liparis campylostalix* Rchb.f.	Jian Xue Qing	Orchidaceae	Whole plant	Decoction, alcohol maceration	Internal lesion caused by overexertion	NY-015		0.02	0.02
*Lonicera maackii* (Rupr.) Maxim.	Jin Yin Hua	Caprifoliaceae	Flowers	Decoction	Clear and quicken the blood	NY-012		0.04	0.04
*Lysimachia paridiformis* var. *stenophylla* Franch.	Zhui Feng San	Primulaceae	Whole plant	Bath	Rheumatism	NY-061	B	0.10	0.10
*Lysionotus pauciflorus* Maxim.	Shi Yang Mei	Gesneriaceae	Whole plant	Decoction	Rheumatism	NY-077	B	0.04	0.04
*Magnolia officinalis* Rehd. et Wils.	Hou Po Guo	Magnoliaceae	Fruits	Decoction	Suppress cough	NY-007	A	0.02	0.02
*Mahonia oiwakensi* Hayata	Ci Huang Lian	Berberidaceae	Roots	Decoction	Rheumatism, internal lesion caused by overexertion	NY-027		0.04	0.04
*Millettia pachycarpa* Benth.	Ku Tan Zi	Fabaceae	Seeds	Powder with boiled water	Abdominal pain	NY-008		0.02	0.02
*Myrica nana* A. Chev.	Suan Yang Mei	Myricaceae	Root barks,Leaves	Decoction, raw	Diarrhea, hematochezia	NY-256		0.04	0.04
*Nandina domestica* Thunb.	Nan Tian Zhu	Berberidaceae	Stems, leaves, flowers	Alcohol maceration, bath	Swelling	NY-065	B	0.02	0.02
*Neolepisorus fortune* Li Wang	Shan Ji Wei	Polypodiaceae	Whole plant	Decoction	Anti-inflammatory	NY-083		0.04	0.04
*Pachysandra axillaris* subsp. *stylosa* (Dunn) Boufford & Q.Y. Xiang	Shan Ban Deng	Buxaceae	Whole plant	Decoction	Icterohepatitis	NY-108		0.08	0.08
*Panax japonicus* (T.Nees) C.A.Mey.	Ma Ye San Qi	Araliaceae	Roots	Alcohol maceration, decoction	Internal lesion caused by overexertion, nourishing	NY-107	A	0.04	0.04
*Paris polyphylla* Sm	Du Jiao Lian	Melanthiaceae	Rhizomes	Decoction, alcohol maceration	Cardiopathy, sores, swelling	NY-144	AB	0.13	0.13
*Perilla frutescens* (L.) Britton	Zi Su Ye	Lamiaceae	Aerial parts	Decoction	Cold	NY-133		0.04	0.04
*Periploca forrestii* Schltr.	Hei Gu Teng	Apocynaceae	Vines	Decoction	Rheumatism, lumbago and leg pain	NY-251	B	0.04	0.04
*Peucedanum praeruptorum* Dunn	Yi Ma Cai	Apiaceae	Whole plant	Decoction	Diaphoresis	NY-067	A	0.06	0.06
*Phellodendron chinense* var. *glabriusculum* C.K.Schneid.	Huang Guo Pi	Rutaceae	Barks	Decoction	Heat-clearing and dampness-drying	NY-252		0.04	0.04
*Phytolacca acinosa* Roxb.	Da Han Cai	Phytolaccaceae	Rhizomes	Decoction	Hydronics, nourishing	NY-036	A	0.04	0.04
*Plantago major* L.	Che Qian	Plantaginaceae	Whole plant	Decoction	Heat-clearing and detoxifying	NY-073		0.13	0.13
*Pleione yunnanensis* (Rolfe) Rolfe	Hong Ji	Orchidaceae	Root tubers	Steam with honey	Suppress cough, pulmonary tuberculosis	NY-024		0.04	0.04
*Polygonatum kingianum* Collett & Hemsl.	Lao Hu Jiang	Asparagaceae	Root tubers	Decoction	Icterohepatitis	NY-105	A	0.02	0.02
*Polygonatum punctatum* Royle ex Kunth	Huang Jing	Asparagaceae	Roots	Cook with pork	Nourishing	WangQH032		0.06	0.06
*Polygonum aviculare* L.	Xiao Shui Hong Hua	Polygonaceae	Whole plant	Decoction	Icterohepatitis	NY-085	A	0.02	0.02
*Polygonum capitatum* Buch.-Ham. ex D. Don	Si Ji Hong	Polygonaceae	Whole plant	Decoction	Nephritis	NY-084	B	0.13	0.13
*Polygonum viviparum* L.	Di Ma Feng	Polygonaceae	Rhizomes	Decoction	Dysentery	NY-158		0.04	0.04
*Potentilla discolor* Bunge	Tian Qing Di Bai	Rosaceae	Whole plant	Decoction	Dysentery	NY-163	A	0.13	0.13
*Primula cernua* Franch.	Mi San Hua	Primulaceae	Whole plant	Decoction	Infertility	NY-191		0.04	0.04
*Prunella vulgaris* L.	Bang Chui Cao	Lamiaceae	Whole plant	Decoction	Heat-clearing	NY-119	A	0.04	0.04
*Pyrola calliantha* Andres	Lu Han Cao	Ericaceae	Whole plant	Cook with pork	Rheumatism, suppressing cough, lumbago	NY-020	A	0.06	0.06
*Reineckea carnea* (Andrews) Kunth	Guan Yin Cao	Asparagaceae	Whole plant	Decoction, alcohol maceration	Cold, relax the veins and stimulate blood circulation	NY-264	B	0.06	0.06
*Rhodiola yunnanensis* (Franch.) S. H. Fu	Hu Dou Qi	Crassulaceae	Roots	Alcohol maceration	Internal lesion caused by overexertion	NY-016		0.02	0.02
*Rubia cordifolia* L.	Xiao Hong Teng	Rubiaceae	Rhizomes	Alcohol maceration	Relax the veins and stimulate blood circulation	NY-253	A	0.04	0.04
*Sambucus javanica* Blume	Wu Yang Cao	Adoxaceae	Whole plant	Bath	Rheumatism	NY-046		0.08	0.08
*Sanguisorba officinalis* L.	Zao Er Huang	Rosaceae	Rhizomes	Decoction	Diarrhea	NY-125	AB	0.02	0.02
*Saxifraga stolonifera* Curtis.	Fan Bei Hong	Saxifragaceae	Whole plant	Ears drop, bath	Earache	NY-094	B	0.04	0.04
*Senecio analogus* DC.	Qian Li Guang	Asteraceae	Aerial parts	Decoction, bath	Heat-clearing, rheumatism	NY-216		0.04	0.04
*Sinocrassula indica* (Decne.) A. Berger	Gou Ya Ban	Crassulaceae	Whole plant	Cook with pork, crush with glutinous rice wine	Mastitis, icterohepatitis	NY-082		0.04	0.04
*Spiraea japonica* L. f.	Yi Cuo Jiao	Rosaceae	Roots	Decoction	Skin disease	NY-260	B	0.06	0.06
*Stellaria media* (L.) Vill	E Er Chang	Caryophyllaceae	Whole plant	Decoction	Lithiasis	NY-116		0.02	0.02
*Talinum paniculatum* (Jacq.) Gaertn.	Hua Qi Shen	Talinaceae	Whole plant	Alcohol maceration, cook with chicken	Nourishing, nose bleeding	NY-050	B	0.02	0.02
*Taraxacum mongolicum* Hand. -Mazz.	Pu Gong Ying	Asteraceae	Whole plant	Decoction	Gynecopathy	NY-128	A	0.08	0.08
*Taxus chinensis* (Pilg.) Rehder	Hong Dou Shan	Taxaceae	Stems,Lraves	Decoction	Suppress cough, relieve pain, gynecopathy	NY-200		0.08	0.08
*Tinospora sagittata* Gagnep.	Shan Ci Ku	Menispermaceae	Root tubers	Powder with boiled water	Neck pain, laryngitis, dysentery, abdominal pain	NY-169	B	0.08	0.08
*Urtica fissa* E. Pritz.	Lao Hu Ma	Urticaceae	Leaves	Bath, decoction	Rheumatism	NY-058		0.08	0.08
*Veratrum nigrum* L.	Xiao Zhong Gen	Melanthiaceae	Rhizomes	Decoction	Detoxification	NY-178	B	0.08	0.08
*Verbena officinalis* L.	Ma Bian Shao	Verbenaceae	Whole plant	Decoction	Varicella	NY-096	A	0.04	0.04
*Viola fargesii* H. Boissieu	Di He Tao	Violaceae	Whole plant	Decoction	Set a bone, palpitation	NY-088		0.08	0.08

## Abbreviations

DBF: Dragon Boat Festival
TCM: Traditional Chinese medicine
UV: Use-value
CI: Cultural importance index
ChP: Pharmacopoeia of the People’s Republic of China
QSG: The Quality Standard of TCM and National Medicine in Guizhou Province
